# Salicylic Acid Regulates Indole-3-Carbinol Biosynthesis Under Blue Light in Broccoli Sprouts (*Brassica oleracea* L.)

**DOI:** 10.3389/fpls.2022.848454

**Published:** 2022-04-05

**Authors:** Tao Wang, Derui Zhang, Boming Yang, Nana Su, Jin Cui

**Affiliations:** ^1^College of Life Sciences, Nanjing Agricultural University, Nanjing, China; ^2^College of Agriculture, Nanjing Agricultural University, Nanjing, China

**Keywords:** broccoli sprouts, blue light, indole-3-carbinol, salicylic acid, gene expression

## Abstract

Indole-3-carbinol (I3C), an important secondary metabolite with strong anti-cancer ability, is widely found in cruciferous plants. Light and phytohormones are one of the most important external and internal signals, respectively, that control the growth, development, and secondary metabolism of the plant life cycle. However, there are few studies about the influence of the blue light and salicylic acid (SA) on the regulation of I3C accumulation. In this study, a negative correlation was found between the content of I3C and SA in different species. Among this, broccoli and *Arabidopsis thaliana* were chosen for further studies. We observed that blue light treatment increased the accumulation of I3C, and exogenous SA treatment significantly inhibited the accumulation of I3C in broccoli sprouts. Based on the RNA sequence, the Kyoto Encyclopedia of Genes and Genomes (KEGG) pathway analysis indicated that blue light promoted the enrichment of differentially expressed genes (DEGs) in plant hormone signal transduction pathways. More specifically, downregulated expression of genes related to SA biosynthesis and upregulated expression of I3C genes related to metabolic pathway were observed under blue light. Taken together, these results suggested that SA negatively regulates blue light-induced I3C accumulation in broccoli sprouts.

## Introduction

Indole-3-carbinol (I3C) produced by the decomposition of indole-3-methyl glucosinolate (I3M) is widely found in many cruciferous vegetables such as collard greens, brussel sprouts, kale, broccoli, cauliflower, cabbage, and turnip (Farag and Motaal, 2010). I3C has been reported as early as the 1970s (Wattenberg, 1977). In the past two decades, numerous studies have proved that I3C is involved in inducing apoptosis and inhibiting cell cycle progression and tumor proliferation (Fan et al., 2006; El-Naga et al., 2014; Choi et al., 2017; Lee et al., 2019). As early, Kushad et al. (1999) found that broccoli contains high content of glucosinolates (GLs), and the content in florets is higher than that in stems and leaves. As the degradation product of GLs, indole-3-methyl isothiocyanate is extremely unstable and almost undetectable. Importantly, I3C has been identified as one of the hydrolysis products of indole-3-methylisothiocyanate (Kokotou et al., 2017). Recent studies have shown that consuming broccoli can effectively prevent the occurrence of cancer owing to the high I3C content in broccoli (Lee et al., 2019). Subsequently, the anti-cancer mechanism of I3C has also been elucidated (Lin et al., 2021). Therefore, I3C is a very promising research hotspot.

Light, an important environmental factor, plays a very important role in regulating plant growth, development, and the accumulation of secondary metabolites. Recent studies have shown that the GLs, a precursor substance for I3C biosynthesis, in cruciferous plants are regulated by light. For example, broccoli sprouts growing under light rather than dark have higher levels of vitamin C, GLs, and phenols (Santiago et al., 2008). Besides, blue light can also regulate plant growth and development and promote the biosynthesis of plant secondary metabolites (Huché-Thélier et al., 2016). It was reported that blue light treatment can stimulate the accumulation of GLs in broccoli and mustard (Pérez-Balibrea et al., 2011; Qian et al., 2016). Meanwhile, Zheng et al. (2018) reported that cabbage treated with 50 μmol m^–2^s^–1^ LED blue light can significantly increase the total GLs content compared with no treatment group. Similarly, blue light can promote the accumulation of aliphatic GLs in broccoli sprouts has also been reported (Xue et al., 2021). In summary, these results indicate that blue light can regulate the accumulation of GLs in plants. However, the regulation of blue light on I3C in cruciferous vegetables has not been investigated. Since blue light can regulate the accumulation of GLs, we speculated that blue light can also regulate the metabolism of I3C in cruciferous plants.

Phytohormones, producing by plant metabolism, regulate various processes of plant growth, development, and environmental adaptation (Nawaz et al., 2017). In *Arabidopsis thaliana*, light promotes the accumulation of ethylene and stimulates growth by inhibiting the stability of phytochrome-interacting factors (PIFs) (Zhong et al., 2012). Besides, Feng et al. (2017) found that light inhibits the rice mesocotyl growth through increasing the accumulation of jasmonic acid and/or decreasing auxin and gibberellin levels based on the results of transcriptome. Particularly, light also regulates abscisic acid-mediated stomata closure in *Vicia faba* guard cells through NO and Ca^2+^ signals (Garcia-Mata and Lamattina, 2007).

Up to now, numerous studies just have focused on several common phytohormones such as auxin, gibberellin, ethylene, and jasmonic acid. However, the report about the effect of light on other phytohormones is still very rare. As a ubiquitous phytohormone in plants, salicylic acid (SA) plays a vital role in regulating plant secondary metabolism. Studies have shown that SA or jasmonic acid can regulate the metabolism of GLs to a certain extent (Yi et al., 2016). In addition, SA treatment regulates the biosynthesis of aromatic, indole, and aliphatic GLs in cruciferous plants (Smetanska et al., 2007). In different cruciferous sprouts, adding SA with an appropriate concentration improves the GLs composition and content by stimulating the biosynthesis of secondary metabolites (Pérez-Balibrea et al., 2011). However, the research on how SA regulates I3C biosynthesis is still unknown.

As a common cruciferous sprouts in our life, broccoli *(Brassica oleracea* L.) has a very significant anti-cancer effect (Lee et al., 2019). Because of its strong anti-cancer ability and low calorie, broccoli sprouts are widely loved by human beings. Unfortunately, I3C content in broccoli is very low although it has significant anti-cancer effects, which resulting in limited anti-cancer ability. Therefore, how to effectively increase the I3C content in cruciferous vegetables and achieve anti-cancer effect through daily diet is an urgent problem to be solved at present. However, the research on how to improve the anti-cancer ability of broccoli sprouts is still very rare. This research mainly used broccoli sprouts as the test material to analyze the effect of SA addition and blue light treatment on the I3C content in broccoli sprouts. This work aims to investigate the potential role of SA participating in blue light to regulate the accumulation of I3C in broccoli sprouts, and explore the mechanism of SA in the regulation of I3C content in broccoli sprouts.

## Materials and Methods

### Plant Materials and Treatments

Broccoli (*B. oleracea* L., Lvyu), leaf mustard (*Brassica juncea* L., Jinsha), and radish (*Raphanus sativus* L., Yanghua) seeds were purchased from Nanjing Jinshengda Seed Co., Ltd. (Nanjing, China). The broccoli heads (Lvyu) were bought from the Suguo Supermarket (Nanjing, China). The seeds were planted according to the method of Wang et al. (2020). After germination, about 150 sprouts of similar size were chosen and transferred to plastic chambers which contained quarter-strength Hoagland’s solution (Wang et al., 2020). Then mustard and radish sprouts were illuminated by LED with white light in a growth chamber (Ningbo Sai Fu Instrument Co., Ltd., Ningbo, China). Besides, the broccoli sprouts were illuminated by LED with white, red and blue light, respectively. The photoperiod was set at 16 h light/8 h dark and the light intensity was set at 200 μmol m^–2^s^–1^. The humidity was set at 75 ± 5%, while the temperature was set at 25 ± 1°C.

After growing in the incubator for 2 days, the broccoli sprouts were treated with SA (Pérez-Balibrea et al., 2011). Firstly, broccoli sprouts were treated with 0, 40, 45, 50, 55, and 60 μM SA to select the suitable dose which has an inhibitory effect on the I3C content of the edible part. Then, the sprouts were divided into four groups, and the following treatments were carried out: sprouts were treated with 10 mL distilled water (Con), only with 10 mL 40 μM SA (SA), only with 10 mL 100 μM Paclobutrazol (a SA biosynthesis inhibitor, Pac), and with 5 mL 40 μM SA and 5 mL 100 μM Pac (SA + Pac), respectively. After 3 days of treatment, the sprouts were collected for measurement.

The *A. thaliana eds5-1* (SALK-091541), *ics1-l1* (SALK-133146), and *ics1-l2* (SALK-093400) mutants were bought from the AraShare.^1^ The *A. thaliana* was cultivated by the method of Wu et al. (2021). Seven days later, the seedlings were transferred to the soil and cultivated for 2 weeks under blue light (200 μmol m^–2^s^–1^).

### Determination of Growth Parameters

Growth parameters [fresh weight (FW), hypotocyl length, and root length] of broccoli, mustard and radish sprouts were determined on three independent experiments (*n* = 3). The growth parameters from five sprouts were determined by a ruler (0.1 cm) and an electronic scale (0.0001 g), respectively. The fresh samples were photographed by camera (Canon, EOS 6D Mark II, Tokyo, Japan).

### Determination of Indole-3-Carbinol Concentration

Indole-3-carbinol concentration was determined by the method described previously (Lee et al., 2010) with some modification. Briefly, 0.5 g edible portion of fresh samples (broccoli, mustard, and radish sprouts) were ground with liquid nitrogen and then the samples were left to autolyze with 8 mL 10 mM phosphate-buffered saline (pH 7.4). At the same time, the sample was incubated for 5 h at 37°C. After that, the crude suspension was centrifuged at 12,500 rpm at 4°C for 10 min, then the supernatant was collected and dried in a Rotary Evaporator (Hualida, LNG-T1OO, Nanjing, China). The residue was extracted twice with 500 μL of ethyl acetate and then dried in a Rotary Evaporator. The residue was dissolved in 1 mL of ethanol. Before the High-performance Liquid Chromatography (HPLC) analysis, the crude extract was filtered with a 0.22-μm filter. Then the extraction liquid was separated by a HPLC system (DIONEX, UltiMate 3000, RS Pump, Milford, CT, United States) with a UV detector on a T3 (ACQUITY UPLC^®^ HSS, 1.8 μm, 2.1 × 100 mm, Milford, CT, United States) column. The parameters were set according to the method of Lee et al. (2010).

### Determination of Salicylic Acid Concentration

Salicylic acid was extracted from edible portion excised from broccoli, mustard, and radish sprouts, and quantified using HPLC as previously described (Lee et al., 2011). 0.5 g samples were used to extract SA. Furthermore, all the methanol was used in this experiment is chromatographic methanol. Besides, all the supernatant was dried in a Rotary Evaporator (Hualida, LNG-T1OO, Nanjing, China). Finally, the residue was dissolved with 600 μL 40% chromatographic methanol. Before the HPLC analysis, the crude extract was filtered with a 0.22-μm filter. Then the extraction liquid was separated by a HPLC system with a UV detector on a C18 (Rapid Resolution HD, 2.1 × 50 mm, 1.8-Micron) column. The parameters were set according to the method of Lee et al. (2011).

Besides, as a key enzyme for the synthesis of SA, the activity of the phenylalanin ammonia-lyase (PAL) in broccoli sprouts were measured by the method of Lister et al. (2015).

### Transcriptome Sequencing

The total RNA was extracted from broccoli sprouts treated with white, red, and blue light (W, R, B), respectively, with three biological replicates using RNA extraction kit by the manufacturer’s instructions (Invitrogen, Gaithersburg, MD, United States). And each biological replicate composed of a pool of sprouts. Then the qualified RNA was sent to Genedenovo Biological Co., Ltd. (Guangzhou, China) for RNA sequencing (RNA-seq) on the illumina sequencing platform. To ensure data quality, FASTP is used for quality control of raw reads, filtering low quality data and obtaining clean reads (Chen et al., 2018).

Then the clean reads aligned to the *B. oleracea* (wild cabbage^2^) reference genome. DEGs were defined as genes with |log_2_FC| ≥ 1 and *P*-adjust < 0.05. The DESeq2 software was used to analysis DEGs between pairwise comparison (Love et al., 2014). The fragments per kilobase of transcript per million mapped reads (FPKM) can be directly used to compare gene expression differences between different samples. The Gene Ontology (GO) and the Kyoto Encyclopedia of Genes and Genomes (KEGG) of the differentially expressed genes (DEGs) were analyzed with an online tool.^3^

### Quantitative Reverse Transcription PCR Analysis

Five-day-old broccoli sprouts with different treatments (0.5 g) were collected and the RNA was extracted by RNA extraction kit (Invitrogen, Gaithersburg, MD, United States). Single-stranded cDNA synthesis was carried out with total RNA using the reagent kit (TOYOBO CO., LTD., Osaka, Japan). Next, the Mastercycler^®^ ep realplex real-time PCR system (ABI7500, MD, United States) system was used to carry out the reaction in a system of 20 μL (10 μL 2 × ChamQ Universal SYBR qPCR Master Mix, 0.5 μL forward/reverse primer, 9 μL cDNA). The program was used by the method of Wu et al. (2021). The Actin was used for a housekeeping gene and the transcription level of these genes were calculated by the 2^–ΔΔCt^ method (Livak and Schmittgen, 2001). Primers were designed by National Center for Biotechnology Information (NCBI), and the primer sequences were listed in Supplementary Tables 1, 2. Each cDNA sample was run in triplicate.

### Verification of Homozygous in *Arabidopsis thaliana*

*Arabidopsis thaliana* DNA was extracted by the manufacturer’s instructions (Genenode Solution for Life science, Beijing, China). Then the leaves of *ics*1*-L1*, *ics*1*-L2*, *eds5*, and wild type (WT) seedlings were collected and the homozygosity was detected through PCR according to the method of Wu et al. (2021). The primers were designed by online website.^4^ Besides, the primers sequences were listed in Supplementary Table 3.

### Statistical Analysis

All experimental data were analyzed by the SPSS (Chicago, IL, United States). The results were shown as the means ± standard deviation (SD) of three independent experiments. The difference between the treatments were tested by analysis of variance (ANOVA) combined with Duncan’s multiple range, *P* < 0.05 represents the significant difference between the treatments. R package (version 4.0.5)^5^ was used for Pearson correlation analysis (Core et al., 2015). The graph was made by GraphPad.^6^

## Results

### Plant Growth Parameters Among Different Species

First we compared growth parameters among different species. The fresh weight (FW) of broccoli and radish sprouts were higher than mustard sprouts (Supplementary Figure 1B). Compared with broccoli sprouts, the hypocotyl length of radish and mustard sprouts were higher (Supplementary Figure 1C). However, among the three species, broccoli sprouts have the longest root length (Supplementary Figure 1D).

### Indole-3-Carbinol and Salicylic Acid Content Between Different Species

Subsequently, we explored the changes in I3C and SA contents among the three species. As shown in Supplementary Figure 1E, the I3C content in broccoli sprouts was 28.5 and 29.8% higher than mustard and radish sprouts, respectively. By contrast, the content of SA in different species was just the opposite. In all species, broccoli sprouts presented the lowest SA concentration, while the content of mustard and radish sprouts were higher. There were no differences in SA and I3C content between mustard and radish sprouts (Supplementary Figure 1F). Since broccoli sprouts had the highest content of I3C, it would be used in subsequent experiments. In order to further verify whether there were differences in I3C content of broccoli sprouts during different periods, the I3C content was detected using HPLC (Supplementary Figure 2). The I3C content of sprout stage was 43% higher than mature stage (Supplementary Figure 2B). Therefore, in this study, broccoli sprouts would be used in the next experiments.

### The Influence of Different Light on Indole-3-Carbinol and Salicylic Acid Content and Growth Parameters

**FIGURE 1:**
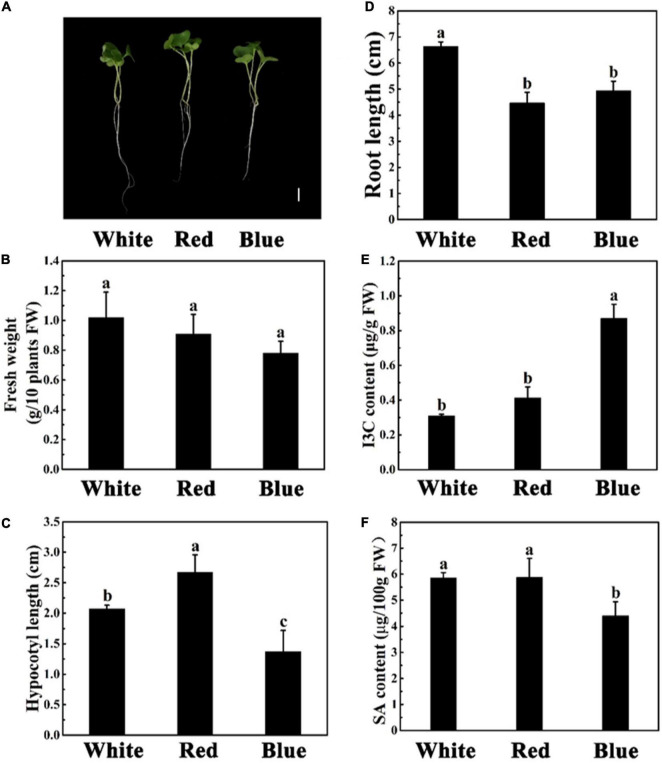
Phenotypic parameters, I3C content, and SA content of different sprouts. Phenotypes of 5-days-old mustard, radish, and broccoli sprouts grown under white light (200 μmol m^–2^s^–1^). **(A)** Phenotypes of 5-days-old mustard, radish, and broccoli seedlings grown under white light. Bar = 1 cm. **(B–D)** The fresh weight **(B)**, hypocotyl length **(C)**, and root length **(D)** after 5 days of growth. **(E,F)** Effects of different species of sprouts on the content of I3C **(E)** and SA **(F)**. Data represented as means ± SD from three independent experiments. Different letters indicated statistical differences (*P* < 0.05).

Light, one of the key environmental factors, regulates plant growth and metabolism. Next, we explored the effect of different light on the phenotype, I3C content, and SA content of broccoli sprouts (Figure 1). Effects of light on plant growth, biomass, and root length of broccoli sprouts were analyzed (Figure 1). The FW, hypocotyl length, and root length of broccoli sprouts were 17.4, 24.6, and 34.6% lower in the blue light treatment than white light treatment, respectively (Figures 1A–D). In addition, the hypocotyl of broccoli sprouts was the longest under red light. However, there were no significant differences in FW and root length under red and blue light treatments (Figures 1A–D).

What’s more, according to the Figure 1E, we found that blue light treatment significantly increased the content of I3C. In contrast, the SA content was the lowest under blue light (Figure 1F), which was consistent with our suppose. Interestingly, there were no difference in I3C and SA contents under white and red light treatments.

### Effects of Salicylic Acid Treatment on Indole-3-Carbinol Content

In order to further verify our conjecture, we explored the influence of exogenous SA on I3C content. In order to determine the appropriate concentration of SA, we conducted a screening experiment for the concentration of SA (Supplementary Figure 3). As shown in Supplementary Figure 3, exogenous addition of SA could significantly reduce the content of I3C as compared to the control (Supplementary Figure 3B). Compared with the control, the content of I3C decreased by 51.7, 41.1, 43.4, 37.7, and 40.6% under 40, 45, 50, 55, and 60 μM SA treatments, respectively. Considering that excessive SA treatment may affect the growth and metabolism of plants, we selected 40 μM SA for subsequent analysis.

**FIGURE 2:**
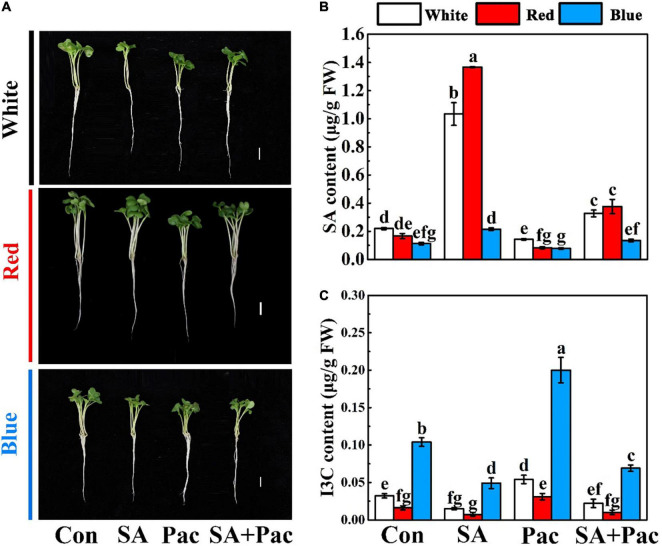
Experiment of exogenous SA treatment. **(A)** The phenotypes under white light, red light, and blue light, respectively. The effect of SA, Pac, and their combination treatments on phenotype under different light. 3-days-old sprouts grown hydroponically were treated with nutrient solution only (Con), 40 μM SA, 100 μM Pac, 40 μM SA + 100 μM Pac (SA + Pac) for 3 days. Bar = 1 cm. **(B,C)** The SA **(B)** and I3C **(C)** content of broccoli sprouts under white light, red light, and blue light, respectively. Data represented as means ± SD from three independent experiments. Different letters indicated statistical differences (*P* < 0.05).

Next, we analyzed the influence of exogenous SA on endogenous SA and I3C content. As shown in Figure 2A, under the same light treatment, there was no significant difference in the growth parameters of broccoli sprouts in SA experiment (Figure 2A). The content of SA significantly decreased by sole Pac treatment compared with that of the control. Besides, the content of SA was elevated with SA + Pac co-treatment, as compared with that of the sole Pac treatment (Figure 2B). In contrast, low I3C content could be observed in broccoli sprouts, after SA treatment. However, compared with control, Pac treatment alone significantly increased the content of I3C. Compared with sole Pac treatment, I3C content was significantly reduced in the co-treatment (Figure 2C).

### Functional Identification of the Differentially Expressed Genes

**FIGURE 3:**
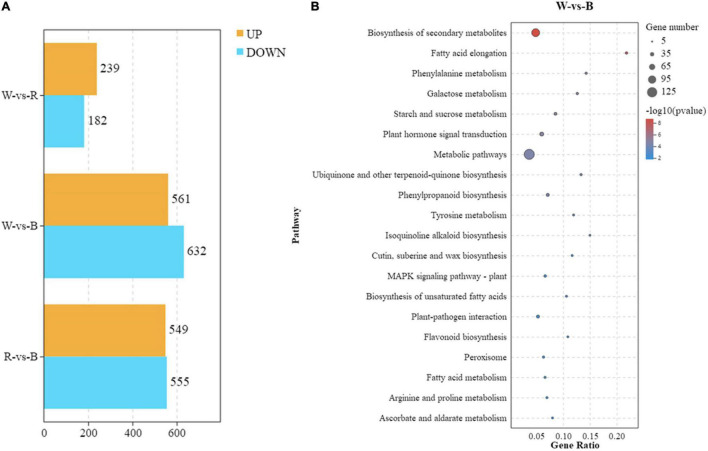
Analysis of transcriptome data and SA transcription level. **(A)** Numbers of DEGs between different light treatments. **(B)** KEGG analysis between W-vs.-B treatment groups. Y-axis represents pathways, while x-axis represents Rich Factor. The Rich Factor means the degree of DEGs enrichment in each pathway, the bigger the Rich Factor the greater the DEGs enrichment. Each bubble represents a pathway, and the size of the bubble represents the number of target genes contained in the pathway. Dots of various sizes represent the number of DEGs accumulated in each pathway. The larger the point, the more DEG (*P* < 0.05). The color of the bubble represents the significant degree of enrichment of the pathway.

In order to further reveal the response of broccoli to blue light, RNA-seq analysis of broccoli sprouts treated with different light for 5 days was performed. In our research, we designed three treatment groups including white-vs-red (W-vs-R), white-vs-blue (W-vs-B), and red-vs-blue (R-vs-B), then the DEGs [FDR < 0.05 and |log2(FC)| > 1] were analyzed (Figure 3A). In the W-vs-R group, 482 DEGs were identified with 239 upregulated DEGs and 182 downregulated DEGs. There were 1104 DEGs in the R-vs-B group, among which 549 DEGs were upregulated and 555 DEGs were downregulated. By contrast, blue light induced a large number of DEGs, and the number of DEGs found in the W-vs-B group rose to 1193 including 561 upregulated DEGs and 632 downregulated DEGs.

Gene Ontology analysis is an internationally standardized classification system, which is used to comprehensively describe the function of uncharacterized genes. The DEGs were more related to biological processes, cell components, and molecular functions under different comparison groups (Supplementary Figure 4). Specifically, we observed that the enriched categories between different treatment groups were same, but there were certain differences in the number of DEGs (Supplementary Figure 4). The results of GO analysis indicated that the significantly enriched categories in three comparable groups were “metabolic process,” “single-organism process,” “cellular process,” “binding,” “catalytic activity,” “cell,” “cell part,” and “organelle process.”

Next, we analyzed the top 20 significantly enriched metabolic pathways by KEGG. In the W-vs-R group, the enriched pathways were “metabolic pathways,” “biosynthesis of secondary metabolites,” “phenylpropanoid biosynthesis,” “starch and sucrose metabolism,” and “plant hormone signal transduction” (Supplementary Figure 5A and Supplementary Table 4). A total of 410 DEGs were identified in the R-vs-B group, and these DEGs were mainly enriched in “metabolic pathways,” “biosynthesis of secondary metabolites,” “carbon metabolism,” “starch and sucrose metabolism,” and “glyoxylate and dicarboxylate metabolism” (Supplementary Figure 5B and Supplementary Table 5). However, the DEGs enriched in the W-vs-B group were quite different from other groups. These 439 DEGs were significantly enriched in “metabolic pathways,” “biosynthesis of secondary metabolites,” “plant hormone signal transduction,” “phenylpropanoid biosynthesis,” and “plant-pathogen interaction” (Figure 3B and Supplementary Table 6). These results revealed that the DEGs related to “plant hormone signal transduction” were more enriched in the W-vs-B group.

In order to verify the accuracy of the transcriptome data, 12 DEGs were selected randomly for quantitative reverse transcription PCR (RT-qPCR analysis). The results were very similar to the trend of the RNA-seq data (Supplementary Figure 6), which indicated that the RNA-seq data have a high degree of credibility.

### Expression of Genes Related to Salicylic Acid Biosynthesis Under Different Light

**FIGURE 4:**
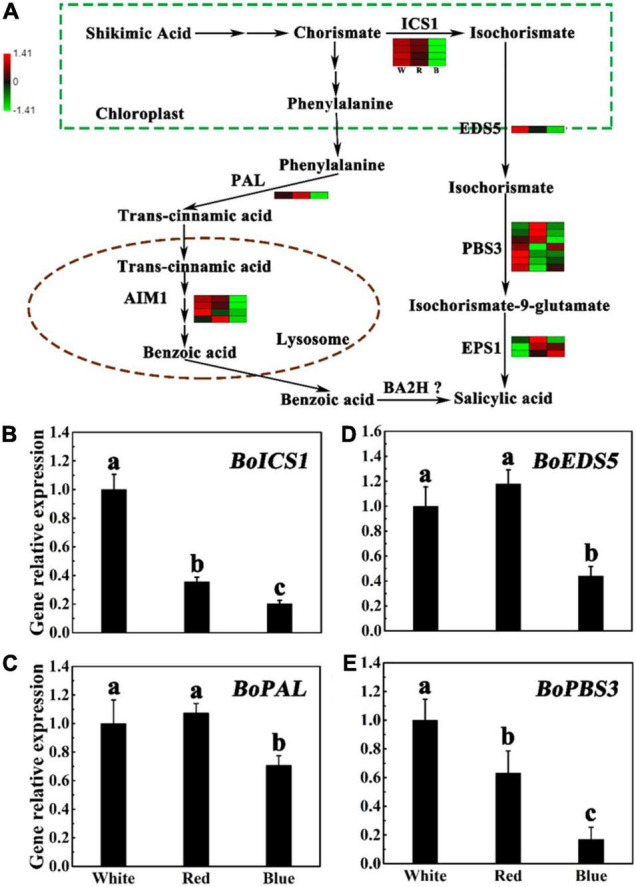
Expression of SA biosynthesis-related genes under different lights. **(A)** Flow chart of SA biosynthesis. The heat map shows the differences in the expression of genes related to I3C synthesis in broccoli sprouts under different lights. W, R, B, means white, red, and blue light. The values of log 2 [fold change (FC)] was represented using the depth of color, with green representing low expression and red representing high expression. Fold change means the ratio of the gene expression in blue light treatment to it in control. **(B–E)** Transcript analysis of SA biosynthesis-related genes in broccoli sprouts with different light treatments. Data represented as means ± SD from three independent experiments. Different letters indicated statistical differences (*P* < 0.05).

Some genes have been reported to be involved in the biosynthesis of SA, such as Mate efflux family protein 5 (*EDS5*), Isochorismate synthase 1 (*ICS1*), Auxin-responsive gh3 family protein3 (*PBS3*), and *PAL* (Rekhter et al., 2019; Torrens-Spence et al., 2019). To explore the role of blue light in SA biosynthesis, the transcript responses of SA metabolism genes to blue light illumination were investigated. According to the transcriptome, we found blue light treatment led to lower gene expression levels of SA biosynthesis pathway, namely, the ICS pathway and the PAL pathway, as compared to control (Figure 4A and Supplementary Table 7). In PAL pathway, the expression level of *ncbi_106300639* encoding PAL was significantly reduced by blue light treatment, and the heat map showed that the levels of FPKM in white and blue light treatment were 2.792 and 1.953, respectively. Besides, four genes encoding AIM1 were also downregulated under blue light treatment. We further compared genes in ICS pathways according to transcriptome by heat map (Figure 4A and Supplementary Table 7), and founded four genes (encoding ICS1) and one gene (encoding EDS5) were remarkably downregulated under blue light treatment compared with other treatments, but there were no significantly differences in the seven genes (encoding PBS3) and three genes (encoding EPS1). Taken together, these results highlight that blue light remarkably affects the expression of genes implicated in SA biosynthesis processes in broccoli sprouts. In particular, in PAL pathway. However, the genes encoding benzoic acid 2-hydroxylase (BA2H) in plants have not yet been resolved (Leon et al., 1993). Compared with white light treatment, RT-qPCR analysis confirmed that the transcript levels of *BoEDS5*, *BoICS1*, *BoPBS3*, and *BoPAL* were obviously downregulated under blue light, which is consistent with the RNA-seq data. Under red light treatment, with the exception of *BoEDS5*, these genes are slightly downregulated in comparison with the white light treatment (Figures 4B–E). Interestingly, there were no significant difference in the level of transcription of these genes between white and red light treatments, as compared with that of the blue light treatment (Figure 4).

### Effects of Different Light on Phenylalanin Ammonia-Lyase Enzyme Activity and Gene Expression Level

Previous studies have shown that PAL plays an important role in the biosynthesis of SA (Huang et al., 2010). Therefore, we further analyzed the activity of PAL under various light conditions. We found that the activity of PAL was significantly reduced under blue light compared with white light and red light (Supplementary Figure 7).

### Expression of Genes Related to Indole-3-Carbinol Biosynthesis Under Different Light

**FIGURE 5:**
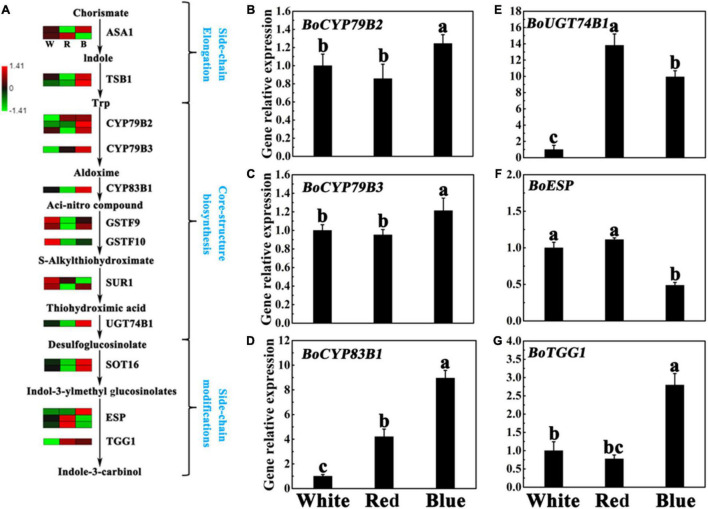
Expression of I3C synthesis-related genes under different lights. **(A)** Flow chart of I3C biosynthesis. The heat map shows the differences in the expression of genes related to I3C synthesis in broccoli sprouts under different lights. The values of log 2 [fold change (FC)] was represented using the depth of color, with green representing low expression and red representing high expression. Fold change means the ratio of the gene expression in blue light treatment to it in control. **(B–G)** Transcript analysis of I3C biosynthesis-related genes in broccoli sprouts with different light treatments. Data represented as means ± SD from three independent experiments. Different letters indicated statistical differences (*P* < 0.05).

**FIGURE 6:**
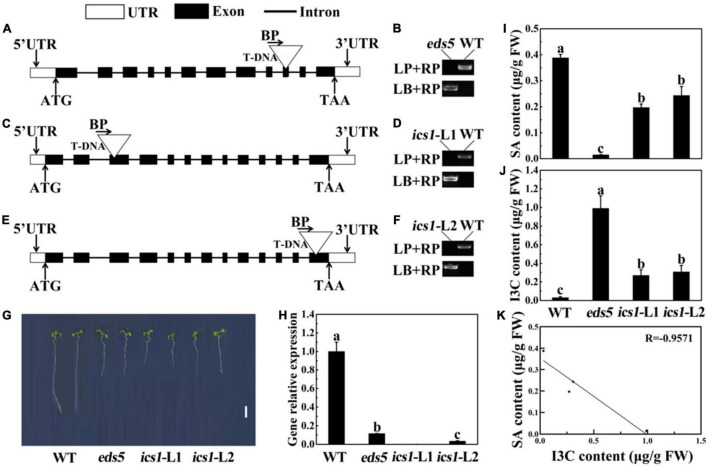
Verification of SA mutants in *Arabidopsis thaliana*. **(A,C,E)** Schematic representation of the eds5 **(A)**, ics1-l1 **(C)**, and ics1-l2 **(E)** gene showing the insertion location of T-NDA. **(B,D,F)** Verification of homozygosity in *Arabidopsis thaliana* mutants. LP, left genomic primer; RP, right genomic primer; BP, T-DNA border primer; LB, the left T-DNA border primer. **(G)** Phenotype of seedlings grown for 7 days. Bar = 0.5 cm. **(H)** Expression levels of related genes in *Arabidopsis thaliana* wild-type and mutants. **(I)** SA content in wild type and mutant. **(J)** I3C content in wild type and mutant. **(K)** Correlation analysis between SA content and I3C content in *Arabidopsis thaliana*. Data represented as means ± SD from three independent experiments. Different letters indicated statistical differences (*P* < 0.05).

Indole-3-carbinol is produced by the hydrolysis of I3M (Esteve, 2020). Besides, there are many genes involved in the production of I3C, including *CYP79B2* (Cytochrome p450, family 79, subfamily b, polypeptide 2), *CYP79B3* (Cytochrome p450, family 79, subfamily b, polypeptide 3), *CYP83B1* (Cytochrome p450, family 83, subfamily b, polypeptide 1), *UGT74B1* (Udp-glucosyl transferase 74b1), *SOT16* (cytosolic sulfotransferase 16), *TGG1* (Thioglucoside glucohydrolase 1), and *ESP* (Epithiospecifier protein), etc (Zang et al., 2010). As a comparative analysis of DEGs in different treatments is very important to explore key genes in I3C biosynthesis regulation, we further compared genes in I3C pathways according to the FPKM by heat map (Figure 5A), and revealed vital genes took part in I3C metabolism, as listed in Supplementary Table 8. As shown in Figure 6A, the genes involved in side-chain elongation stage, namely, *BoTSB1* (tryptophan synthase beta-subunit 1) and *BoASA1* (anthranilate synthase alpha subunit 1) showed higher levels in blue light treatment than other treatments. The core-structure biosynthesis stage is the important pathway in the biosynthesis of I3C, and the key genes in this stage include *CYP79B2/B3*, *CYP83B1*, *GSTF9/10* (glutathione S-transferase F9/10), *SUR1* (S-alkyl-thiohydroximate lyase SUR1), and *UGT74B1* (Grubb and Abel, 2006). Under blue light treatment, most of these genes were upregulated compared with other light treatments. Noticeably, the expression level of *ncbi_106328484* (encoding GSTF10) was significantly downregulation. As for the side-chain modifications stage, we found two genes (encoding STO16) and one gene (encoding TGG1) were upregulated under blue light treatments. However, the two genes, namely, *MSTRG.17449* and *ncbi_106306884* (encoding ESP) were lower in blue light treatment than that in other light treatments (Figure 5A and Supplementary Table 8). These results indicate that the upregulation of genes may play an important role in I3C metabolism.

To determine how blue light is involved in the regulation of I3C metabolism in the cells, the RT-qPCR assay was performed to analyze the transcript levels of four GLs biosynthesis genes, namely, *CYP79B2, CYP79B3, CYP83B*, and *UGT74B1*, and the transcript levels of two major GLs-degrading genes, namely, *ESP* and *TGG1*, in the edible part of broccoli sprouts. The results confirmed that the relative expression of *BoCYP79B2/B3, BoCYP83B1*, and *BoUGT74B1* were significantly upregulated under blue light (Figures 5B–E). Interestingly, these genes had similar expression levels under white light and red light except *BoUGT74B1* and *BoCYP83B1* (Figure 5). Meanwhile, compared with the control, the gene expression of *BoTGG1* involved in the breakdown process had a higher level under blue light (Figure 5G). However, blue light treatment significantly decreased the expression level of *BoESP* as compared to other light treatments (Figure 5F). Interestingly, in contrast, the transcript levels of these genes did little respond to red light illumination.

### Verification of Salicylic Acid Biosynthesis Mutants in *Arabidopsis thaliana*

*Arabidopsis thaliana*, the model plant of the cruciferae family, can also produce I3C through secondary metabolism processes. Therefore, in order to further verify the negative regulatory effect of SA in the process of I3C biosynthesis, we used *A. thaliana* wild-type (WT) and the SA biosynthesis defective mutants (*eds5*, *ics1-l1*, and *ics1-l2*) for subsequent analysis. Mutations in these genes cause a decrease in SA accumulation (Rekhter et al., 2019; Torrens-Spence et al., 2019). The homozygous verification of *A. thaliana* indicated that all the *A. thaliana* mutants in our experiment were homozygous, which was conducive to our further experimental analysis (Figures 6A–F). In this research, we found that *eds5*, *ics1-l1*, and *ics1-l2* seedlings displayed shorter hypocotyl and root length compared with WT (Figure 6G). Next, we used RT-qPCR to further verify the expression of these mutant genes. As shown in Figure 6H, RT-qPCR analysis confirmed that the expression of *AtEDS5* and *AtICS1* was significantly downregulated in mutants, compared with WT, which supported the loss of function of these genes in the mutant.

Next, we used *A. thaliana*, which grown under blue light for 2 weeks, to detect SA and I3C content. The results showed that in contrast to the WT, the SA content in the mutant was significantly reduced, and the *eds5* had the lowest SA content (Figure 6I). Compared with the WT, the content of SA in *eds5*, *ics1-l1*, and *ics1-l2* was reduced by 96, 49, and 37%, respectively (Figure 6I). At the same time, the content of I3C was just the opposite of the SA content (Figure 6J). In contrast to *ics1-l1*, *ics1-l2*, and WT, the I3C content was highest in *eds5* sprouts (Figure 6J). However, compared with WT, *eds5*, *ics1-l1*, and *ics1-l2* effectively elevated the content of I3C by 97, 88, and 90%, respectively (Figure 6J). According to correlation analysis, we found that SA and I3C were negatively correlated, and the correlation coefficient was −0.9574 (Figure 6K).

## Discussion

In the past 5 years, I3C has been widely studied as an important anticancer substance (Kokotou et al., 2017; Lee et al., 2019; Lin et al., 2021). Previous researchers have found that blue light increases the accumulation of GLs in broccoli sprouts (Pérez-Balibrea et al., 2011; Xue et al., 2021). However, the effect of blue light on the accumulation of I3C, the degradation product of GLs, in broccoli is still not deeply analyzed. In this study, the effect of blue light on the I3C content was discussed *via* analyzing the morphology, SA content, and I3C content of different cruciferous plants. Meanwhile, we also explored the role of SA in blue light-induced I3C accumulation using physiological methods and transcriptomic techniques.

In recent decades, sprouts with high nutrition such as broccoli and radish sprouts have been deeply loved by consumers. In this study, we first used the sprouts of common cruciferous vegetables (mustard, radish, and broccoli) to explore the phenotypes of different species as well as the differences in I3C and SA contents (Supplementary Figure 1). The results indicated that the contents of I3C and SA in different species showed negative correlation. The same phenomenon was also observed in *A. thaliana*, the accumulation of I3C in *eds5*, *ics1-l1*, and *ics1-l2* mutants resulted from the reduction of SA content (Figure 6). Similarly, previous studies have shown that SA treatment can inhibit the content of indole GLs in *A. thaliana* (Gigolashvili et al., 2008). In addition, among these cruciferous vegetables, broccoli sprouts had the highest content of I3C and the lowest content of SA (Supplementary Figures 1E,F). At the same time, we found that the content of I3C in broccoli sprouts was significantly higher than that in mature stage (Supplementary Figure 2B), which is very important for the research of sprouts. Interestingly, similar results have been reported by Fahey et al. (1997) who found that the content of GLs in mature broccoli is about 1 ∼ 10% of sprouts. Coincidentally, previous researchers have also found the similar phenomenon in *Brassica nigra* and *B. juncea* (Rangkadilok et al., 2002). All these results indicated that sprouts have the higher GLs content than mature stage.

As an energy source for photosynthesis and a key environmental factor, light plays an important role in the regulation of secondary metabolism in plants. In particular, numerous studies have reported that blue light is able to regulate the secondary metabolism in different plants (Pérez-Balibrea et al., 2011; Qian et al., 2016). For example, previous reports indicated that blue light induces the accumulation of vitamin C, total phenols, and GLs (Pérez-Balibrea et al., 2011). However, there is no report on the role of blue light in regulating I3C accumulation. Therefore, we explored the effect of different light on the content of I3C in broccoli sprouts. Our results showed that blue light significantly increased the content of I3C (Figure 1E), while the content of SA under blue light was significantly reduced compared with other light conditions (Figure 1F), which are consistent with a recent study (Zheng et al., 2018). Coincidentally, Kopsell and Sams’s (2013) study indicated that blue light treatment improves the content of GLs. These results indicated that blue light is very effective for the accumulation of I3C in broccoli sprouts. On the contrary, many studies have suggested that blue light inhibits the content of GLs. For example, Qian et al. (2016) found that the content of GLs in Chinese kale sprouts was inhibited by blue light. Besides, blue light treatment had no significant effect on the content of GLs in *Brassica napus* L. (Park et al., 2019). Therefore, we speculate that the difference in the effect of blue light on GLs may be caused by the distinct mechanisms in response to blue light in diverse plants. Because of these conflicting results, the role of blue light in improving I3C content requires more evidence.

As an important phytohormones, SA has been regarded as the central regulator of GLs biosynthesis in recent years (Gigolashvili et al., 2008), but the regulation of I3C still remains unknown. In our current research, we treated broccoli sprouts with exogenous SA with different concentrations and found that the I3C content was significantly inhibited under SA treatment, compared with the control (Figure 2 and Supplementary Figure 3). What’s more, the results of exogenous SA and its inhibitor Pac treatment experiments indicated that SA can negatively regulate the accumulation of I3C (Figures 2B,C). It is noteworthy that 50 μM SA treatment for 3 days significantly inhibited the content of indole GLs in broccoli sprouts (Pérez-Balibrea et al., 2011). More importantly, exogenous SA treatment can significantly reduce the content of glucoiberin and glucobrassicin (Baenas et al., 2014). In *A. thaliana*, SA inhibits indole GLs biosynthesis through a R2R3 MYB transcription factor, AtMYB28 (Gigolashvili et al., 2008). However, in the early stage of turnip growth, addition 800 mM SA to the culture medium can significantly increase the content of GLs (Smetanska et al., 2007). Therefore, more evidence is needed to explore the reasons behind these contradictory results. In the past 10 years, transcriptomic has been more applied to the study of plant response to light signals due to its powerful gene identification function (Zhou et al., 2011). Based on the transcriptome data of *A. thaliana* under blue light treatment, Wang et al. (2018) found that CRY1 regulates brassinolide signal transduction and photomorphogenesis. In this study, we found that blue light was involved in “plant hormone signal transduction” pathway according to the KEGG enrichment analysis (Figures 3A,B). The RNA-Seq results further indicated that SA might take part in the inhibition of I3C. Importantly, a similar phenomenon was observed in the metabolomics of broccoli sprouts (Xue et al., 2021).

Identification the regulatory network of SA is critical for exploring the I3C metabolism, as well as deepening our understanding of the complex physiological and biochemical processes of cruciferous plants. Numerous genes such as *EDS5*, *ICS1*, and *PBS5* involved in the SA biosynthesis (Rekhter et al., 2019; Torrens-Spence et al., 2019). The RT-qPCR and RNA-Seq results both showed that the expression levels of genes involved in the SA biosynthesis pathway were downregulated under blue light (Figure 4A and Supplementary Table 7). The role of PAL in the biosynthetic pathway of SA has been widely reported (Huang et al., 2010). Therefore, it was very necessary to investigate the function of PAL in broccoli under different light. In this research, we found that the activity of PAL and the gene expression of *BoPAL* were significantly decreased under blue light compared with other light (Supplementary Figure 7). Taken together, these data suggested that these genes were involved in the inhibition of SA biosynthesis under blue light. Interestingly, blue light can promote the accumulation of SA in the photoperiod-sensitive *7B-1* male-sterile mutant (mutation in the *SlGLO2* gene) in *Solanum lycopersicum* (Bergougnoux et al., 2009; Pucci et al., 2017). Similarly, the 150 μmol m^–2^s^–1^ photosynthetically active radiation can promote the accumulation of SA in sunflower hypocotyls (Kurepin et al., 2010). Due to these completely different results, a large number of experiments are also needed to verify the role of light in regulating SA.

The accumulation of I3C in plants is co-regulated by the transcription and translation levels. According to reports, many genes might be involved in the regulation of I3C accumulation in plants such as *CYP79B2*, *CYP79B3*, *CYP83B1*, *UGT74B1 TGG1*, *TGG2*, and *ESP* (Zang et al., 2010). According to the analysis of RNA-Seq and RT-qPCR data, we found the transcript levels of *BoCYP79B2*, *BoCYP79B3*, *BoCYP83B1*, *BoUGT74B1*, and *BoTGG1* were upregulated in the edible part of broccoli sprouts under blue light (Figure 5 and Supplementary Table 8), which indicated that these five genes may be involved in I3C accumulation under blue light. It has been reported that blue light increases the expression of the related genes (*BoMYB28*, *BoCYP79F1*, and *BoCYP83A1*) and affects the biosynthesis of aliphatic GLs (Xue et al., 2021). The same phenomenon was also found in *A. thaliana*, blue light can also enhance the expression of genes related to GLs biosynthesis (Huseby et al., 2013). Taken together, these data indicated that blue light affects I3C and GLs accumulation mainly by regulating the GLs biosynthesis and degradation in broccoli sprouts. However, in order to further understand the clear mechanism by which SA negatively regulates I3C accumulation in broccoli sprouts, more molecular evidence is needed to further explore the interrelationships among these genes.

## Conclusion

In this study, we found that the content of SA and I3C in different cruciferous vegetables showed a negative correlation. Furthermore, blue light can reduce the expression levels of genes related to SA biosynthesis, and then increase the expression levels of genes related to I3C accumulation. Overall, the study revealed that blue light can increase the I3C content by reducing the content of SA.

## Data Availability Statement

The datasets presented in this study can be found in online repositories. The names of the repository/repositories and accession number(s) can be found below: National Center for Biotechnology Information (NCBI) BioProject database under accession number PRJNA798557.

## Author Contributions

NS and JC designed and guided the experiment. TW wrote the manuscript and analyzed the data. DZ modified and submitted the manuscript. DZ and BY provided experimental guidance and ideas. All authors read and approved the final manuscript.

## Conflict of Interest

The authors declare that the research was conducted in the absence of any commercial or financial relationships that could be construed as a potential conflict of interest.

## Publisher’s Note

All claims expressed in this article are solely those of the authors and do not necessarily represent those of their affiliated organizations, or those of the publisher, the editors and the reviewers. Any product that may be evaluated in this article, or claim that may be made by its manufacturer, is not guaranteed or endorsed by the publisher.

## Footnotes

1
https://www.arashare.cn/index/

2
http://www.ncbi.nlm.nih.gov/genome/?term=txid3712[orgn]

3
http://www.omicsmart.com/

4
http://signal.salk.edu/tdnaprimers.2.html

5
https://www.R-Project.org

6
http://www.graphpad-prism.cn
