# Supplementary material for: Salicylic Acid Regulates Indole-3-Carbinol Biosynthesis Under Blue Light in Broccoli Sprouts (Brassica oleracea L.)
Source: Front Plant Sci. 2022 Apr 5;13:848454. doi: 10.3389/fpls.2022.848454 (PMC9016176; doi:10.3389/fpls.2022.848454)
Supplement: Supplementary file 1 [file Data_Sheet_1.DOC]

**
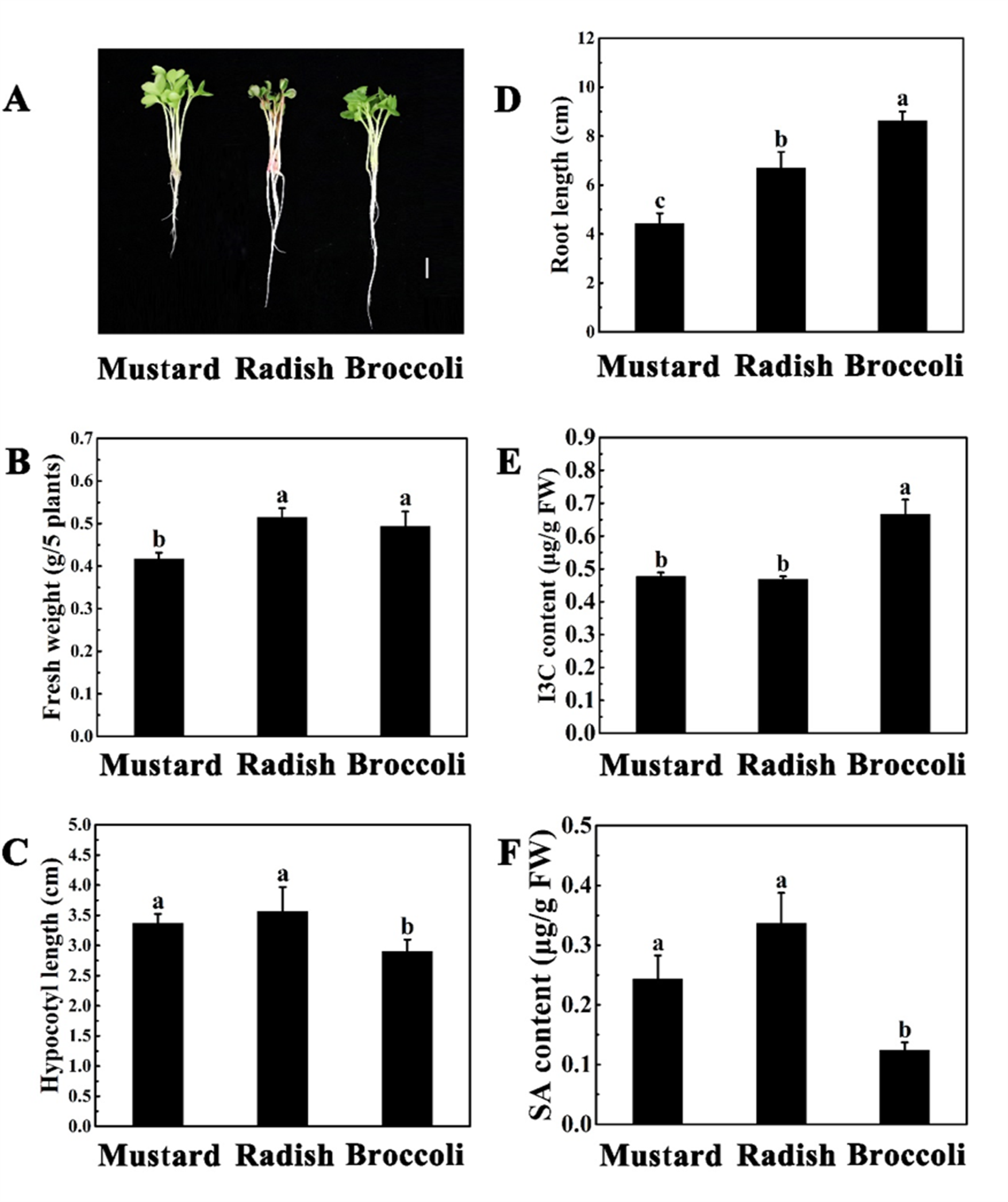
**

**Figure S1** Phenotypic parameters, I3C content, and SA content of different sprouts. Phenotypes of 5-days-old mustard, radish, and broccoli sprouts grown under white light (200 μmol m^-2^ s^-1^). A, Phenotypes of 5-d-old mustard, radish and broccoli seedlings grown under white light. Bar = 1 cm. B, C, and D, The fresh weight (B), hypocotyl length (C), and root length (D) after 5 days of growth. E and F, Effects of different species of sprouts on the content of I3C (E) and SA (F). Data represented as means ± SD from three independent experiments. Different letters indicated statistical differences (P < 0.05).


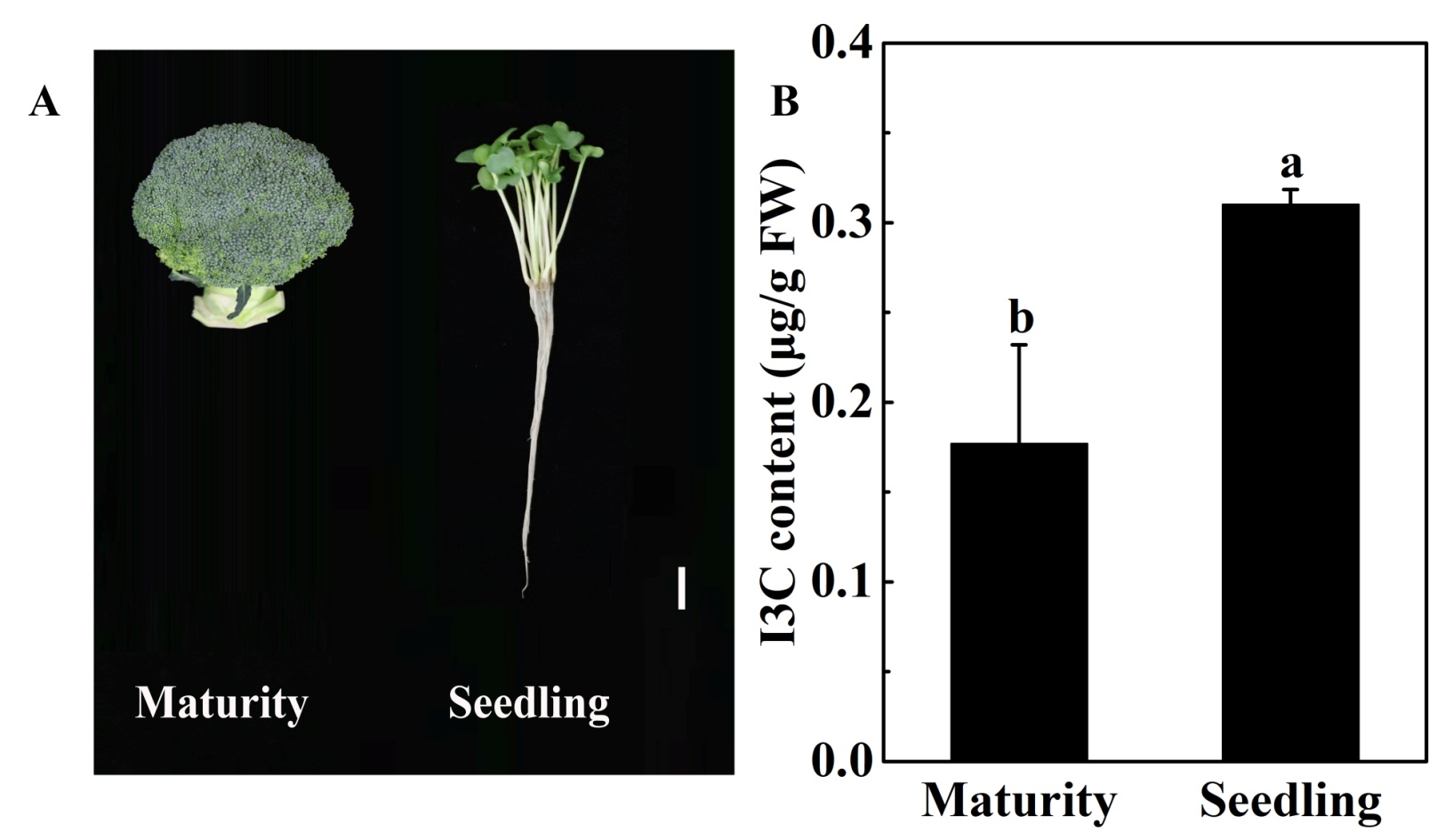


**Fig. S2** Comparison of broccoli at different developmental stages. A, Phenotypes of broccoli in different periods. Bar = 1 cm. B, Effects of different periods on I3C content.


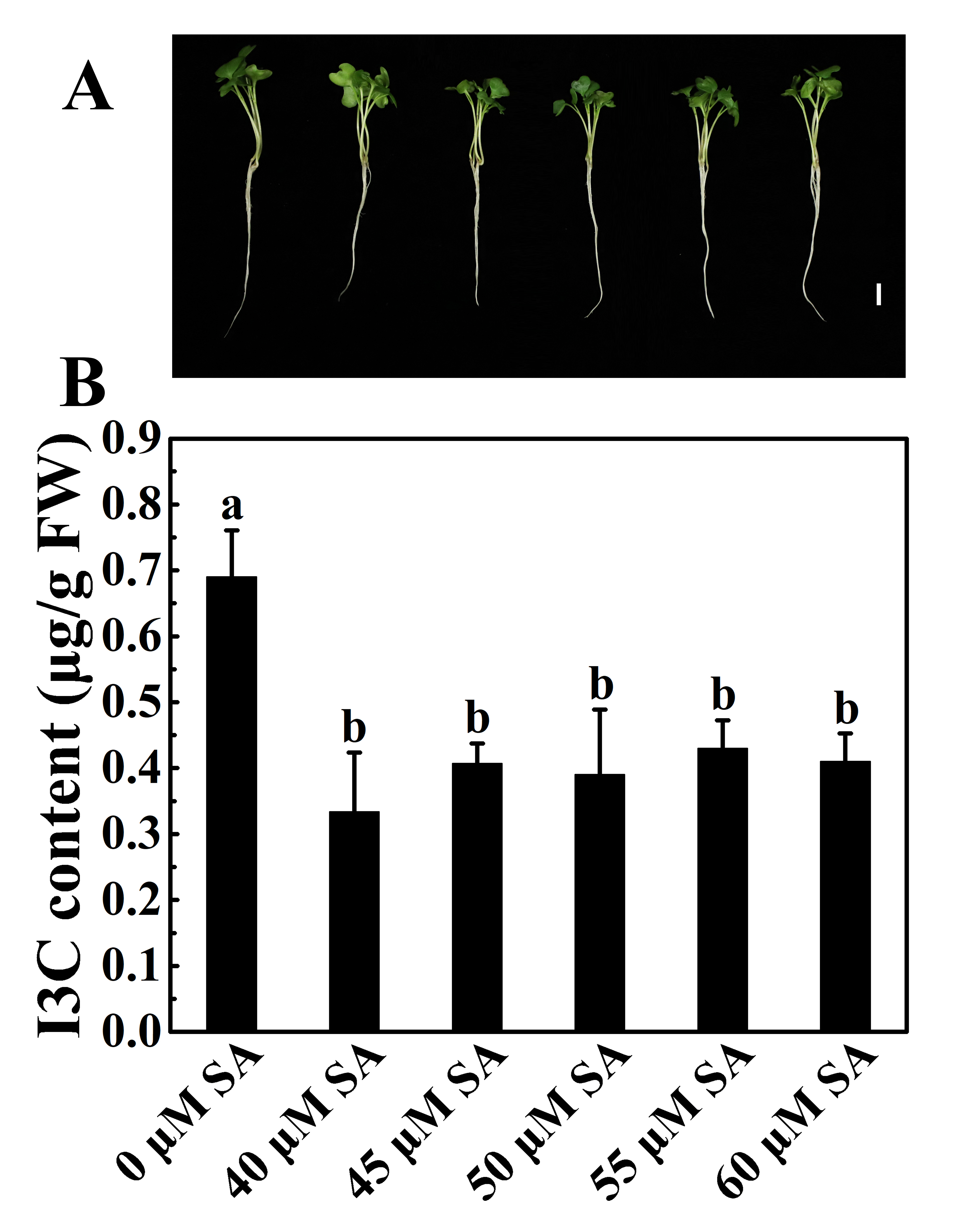


**Figure S3** Experiment of exogenous SA treatment. A, Phenotypes of 3-days-old broccoli sprouts treated with different concentrations of SA (40 μM, 45 μM, 50 μM, 55 μM, and 60 μM) under white light. Bar = 1 cm. B, the content of I3C in broccoli treated with different concentrations of SA under white light. Data represented as means ± SD from three independent experiments. Different letters indicated statistical differences (P < 0.05).


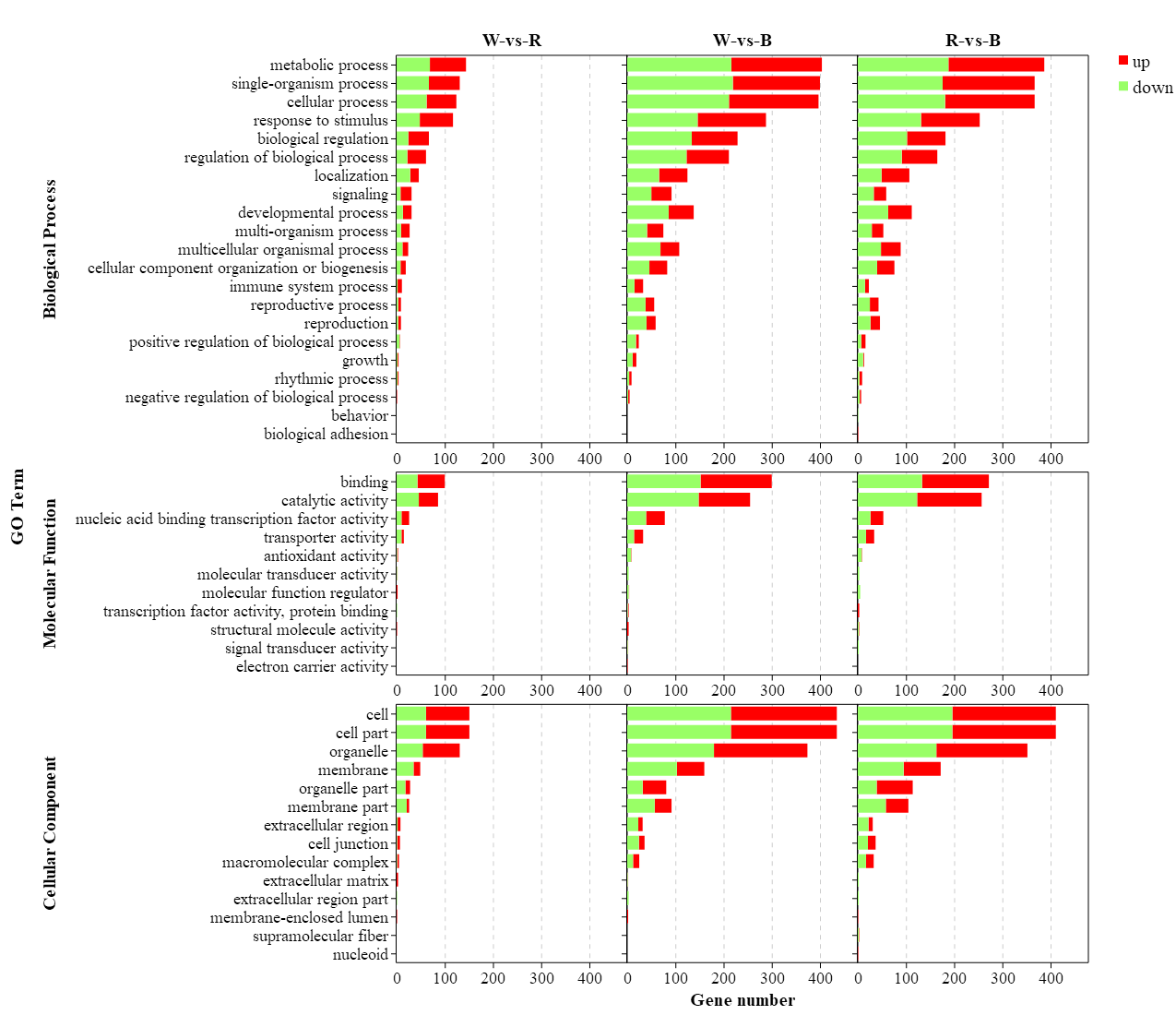


**Fig S4** GO enrichment analysis of DEGs in different treatments. The x-axis represents the number of DEGs number and the y-axis stands for the different GO terms. Annotations are grouped by cellular component, molecular function or biological process based on the Brassica GO annotation information.


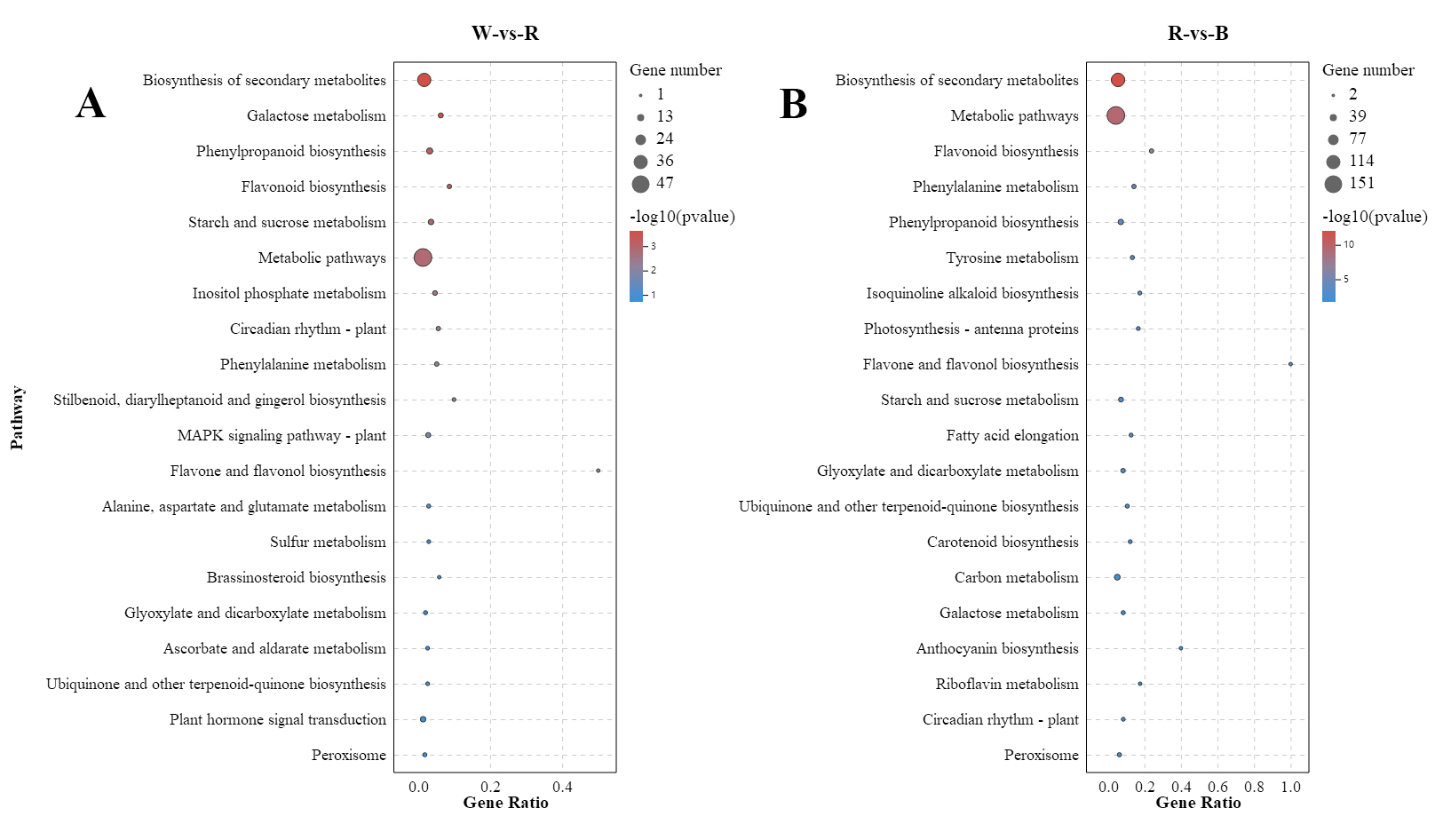


**Fig. S5** KEGG enrichment analysis of different treatments. A, DEGs in W-vs-R treatment; B, DEGs in R-vs-B.





**Fig. S6** RT-PCR validation of the expression of 12 DEGs randomly selected from RNA-seq results. The left Y-axis on behalf of the expression of corresponding genes by RT-PCR analyses and the right Y-axis represents the FPKM of RNA-seq.


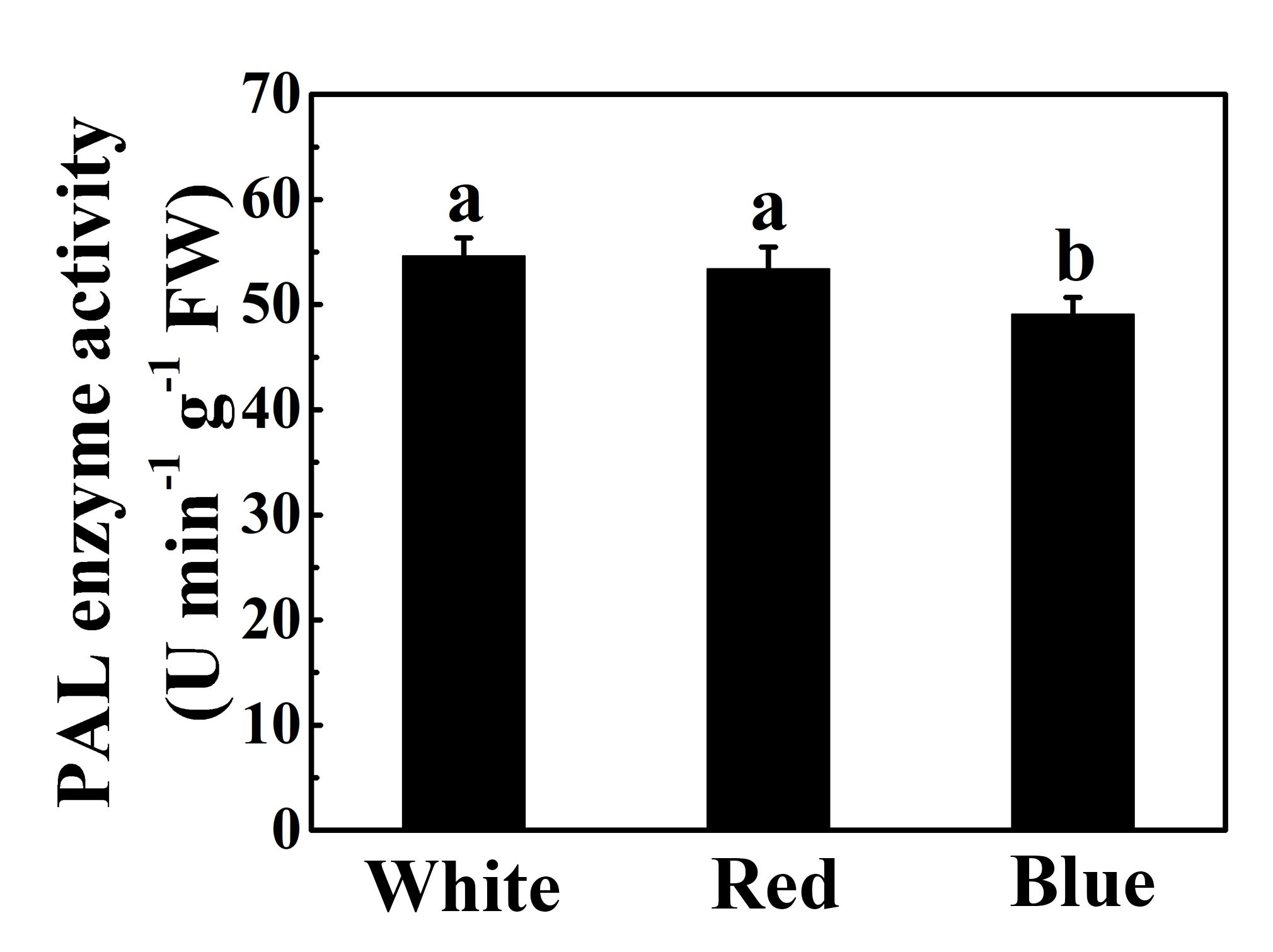


**Figure S7** Effects of different light treatments on enzyme activities related to SA biosynthesis. A, PAL enzyme activity under different light. B, Semi-quantitative level of PAL gene under different light. Data represented as mean SD from three independent experiments. Different letters indicated statistical differences (P < 0.05).
