# Supplementary material for: Salicylic Acid Regulates Indole-3-Carbinol Biosynthesis Under Blue Light in Broccoli Sprouts (Brassica oleracea L.)
Source: Front Plant Sci. 2022 Apr 5;13:848454. doi: 10.3389/fpls.2022.848454 (PMC9016176; doi:10.3389/fpls.2022.848454)
Supplement: Supplementary file 2 [file Data_Sheet_2.doc]

Table S1. Broccoli primers used for RT-qPCR.
Gene	Primer sequence (5' to 3')	
	Forward	Reverse	
Actin	CTGTTCCAATCTACGAGGGTTTCT	GCTCGGCTGTGGTGGTGAA	
BoCYP79B2	CCATTGCTTACCGCTGAT	TCCGACCACTCTGTCTATT	
BoCYP79B3	TTGCTTACCGCTGATGAA	GGATTCTTGGACGAGTCTT	
BoCYP83B1	GGTAAGCGGTATAACGAGTA	CGAGGAGTTCTTGAAGGTAA	
BoESP	GCCTATAACATTGCTGATGG	CACTTGGTCGGACTCATAG	
BoTGG1	GCTATGCTATCGGAACAGA	GTAATCATCACAGGTCCAATC	
BoUGT74B1	GACGCATACTCCGAATCC	GTGAGGTTGTTGGTGAAGA	
BoEDS5	ACCACTTATCCGTTGTCTC	GCTCTTCTTCTCTTCTTCTTC	
BoICS1	GGCTCCATCTCCATCTCT	CCTTCGCATCCGTTCATA	
BoPAL	CTATGGCTTCCTACTGTTCA	CTTGCGTGATGAGATTAGTC	
BoPBS3	AGAACCGTCCATCTAACTG	TCTCACAACCTCGTCTCT	
BoARF32	ACCACAACACTCCCTCGTTG	CGGCGTGTTGTTTCGGTATG	
BoASA1	GCGTCTCTGTATCGCCTGAA	CCAGGCTCAACGGACTCAAA	
BoCYP707A2	ATGGGTTGGCCTTACATCGG	TATTTGTTTTGGCGGGTGGC	
BoGDPD2	GCTCTTCGTACTGTCCTCGT	TACCATTTCCTCTGTGCCCG	
BoGSTF9	TACCGTTCCTGCTGTTGTCG	GTGAGGTTGAGTAGCGGTGG	
BoLEA5	ACAGCTAAGACCCTCTCCGT	CGCTAGACTCTTCCCCAACG	
BoNPR1	ACGCTTCTTCCCACGATGTT	CTCAGGTGTGTCGCCTTCTC	
BoRFS6	TTATGCTGCCTGACTGGGAC	CACTAATGGCCCTAGCGGAG	
BoSOT16	GTTTGTCGGTGTACGGTCCT	CCCATGAACTCAGCCAACCT	
BoSUR1	CCTGTATCGAGGCCGAAGAC	ATGTCGTCCGGCGTTAGTTT	
BoTGA7	ACATTGCAGCAAATGGCGAA	AGAGCACGCAGCCTATGAAG	
BoTSB1	GCGTTTCATTCTCTCGCCAC	CCGTAAGCCTCTCTGCGAAA	


Table S2. Arabidopsis thaliana primers used for RT-qPCR.
Gene	Primer sequence (5' to 3')	
	Forward	Reverse	
Actin	AGGCACCTCTTAACCCTAAAGC	GGACAACGGAATCTCTCAGC	
EDS5	ATCAGGTGATGGCTCAGACG	CGCTGTTCCGATAACTCCCA	
ICS1	GGCGAGGAGAGTGAATTTGC	TGGTAACAGAGAACCATGATGT	


Table S3 Primer sequence for Arabidopsis thaliana homozygous verification.
Gene	LP	RP	LB	
eds5	CGCTGCACCTGTTTTTATCTC	TTCTCCACCGTGTATGGACTC	ATTTTGCCGATTTCGGAAC	
ics1-L1	TCTGATGGATCTCCAATCGTC	GAGATTTCAAGACGCCACTTG		
ics1-L2	TTGAGGTGGAGGGTAAAAAGG	CAGGTACGAGCTTTTGTCCAG		


Table S4. The top 20 metabolic pathways in W-vs-R comparable group.
Top 20 metabolic pathways	Number of DEGs (Total 154)	Pvalue	
Biosynthesis of secondary metabolites	33	3.63	
Galactose metabolism	6	3.54	
Phenylpropanoid biosynthesis	10	3.14	
Flavonoid biosynthesis	4	3.02	
Starch and sucrose metabolism	8	2.89	
Metabolic pathways	47	2.87	
Inositol phosphate metabolism	5	2.46	
Circadian rhythm-plant	4	2.32	
Phenylalanine metabolism	4	2.20	
Stilbenoid, diarylheptanoid and gingerol biosynthesis	2	1.81	
MAPK signaling pathway - plant	6	1.80	
Flavone and flavonol biosynthesis	1	1.72	
Alanine, aspartate and glutamate metabolism	3	1.14	
Sulfur metabolism	2	0.87	
Brassinosteroid biosynthesis	1	0.82	
Glyoxylate and dicarboxylate metabolism	3	0.79	
Ascorbate and aldarate metabolism	2	0.79	
Ubiquinone and other terpenoid-quinone biosynthesis	2	0.79	
Plant hormone signal transduction	8	0.75	
Peroxisome	3	0.71	


Table S5. The top 20 metabolic pathways in R-vs-B comparable group.
Top 20 metabolic pathways	Number of DEGs (Total 410)	Pvalue	
Biosynthesis of secondary metabolites	107	12.02	
Metabolic pathways	151	9.59	
Flavonoid biosynthesis	11	7.17	
Phenylalanine metabolism	11	4.83	
Phenylpropanoid biosynthesis	2	3.91	
Tyrosine metabolism	9	3.83	
Isoquinoline alkaloid biosynthesis	7	3.82	
Photosynthesis - antenna proteins	6	3.23	
Flavone and flavonol biosynthesis	2	3.06	
Starch and sucrose metabolism	16	3.04	
Fatty acid elongation	7	2.95	
Glyoxylate and dicarboxylate metabolism	12	2.94	
Ubiquinone and other terpenoid-quinone biosynthesis	8	2.79	
Carotenoid biosynthesis	6	2.51	
Carbon metabolism	26	2.43	
Galactose metabolism	8	2.15	
Anthocyanin biosynthesis	2	2.08	
Riboflavin metabolism	3	1.89	
Circadian rhythm - plant	6	1.74	
Peroxisome	10	1.72	


Table S6. The top 20 metabolic pathways in W-vs-B comparable group.
Top 20 metabolic pathways	Number of DEGs (Total 439)	Pvalue	
Biosynthesis of secondary metabolites	93	8.72	
Fatty acid elongation	12	7.65	
Phenylalanine metabolism	11	5.13	
Galactose metabolism	12	4.98	
Starch and sucrose metabolism	19	4.96	
Plant hormone signal transduction	34	4.89	
Metabolic pathways	125	4.51	
Ubiquinone and other terpenoid-quinone biosynthesis	10	4.45	
Phenylpropanoid biosynthesis	22	4.39	
Tyrosine metabolism	8	3.33	
Isoquinoline alkaloid biosynthesis	6	3.15	
Cutin, suberine and wax biosynthesis	7	2.91	
MAPK signaling pathway - plant	14	2.69	
Biosynthesis of unsaturated fatty acids	7	2.67	
Plant-pathogen interaction	21	2.51	
Flavonoid biosynthesis	5	2.08	
Peroxisome	10	1.93	
Fatty acid metabolism	9	1.90	
Arginine and proline metabolism 	8	1.86	
Ascorbate and aldarate metabolism	6	1.77	


Table S7. The genes involved in SA accumulation.
Gene	ID	FPKM/W	FPKM/R	FPKM/B	
ICS1	MSTRG.7240	6.743	6.573	5.613	
ICS1	ncbi_106296685	11.2	10.207	5.907	
ICS1	ncbi_106301026	4.29	3.99	3.403	
ICS1	ncbi_106322548	10.213	8.753	4.893	
PAL	ncbi_106300639	2.72	3.19	1.953	
AIM1	MSTRG.7106	0.837	1.09	0.64	
AIM1	ncbi_106303780	0.683	0.517	0.443	
AIM1	ncbi_106304818	39.347	38.803	37.427	
AIM1	ncbi_106312132	6.253	5.973	4.507	
EDS5	ncbi_106327211	2.063	1.14	0.383	
PBS3	MSTRG.7886	0.607	0.44	0.607	
PBS3	ncbi_106295613	1.263	0.87	0.887	
PBS3	ncbi_106318018	10.71	11.467	10.497	
PBS3	ncbi_106319452	9.613	8.82	9.057	
PBS3	ncbi_106319532	5.317	4.383	4.993	
PBS3	ncbi_106336191	1.84	2.01	1.447	
PBS3	ncbi_106342799	0.21	0.19	0.25	
EPS1	ncbi_106292240	0.19	0.287	0.15	
EPS1	ncbi_106311930	10.717	15.657	19.457	
EPS1	ncbi_106340209	0.023	0.077	0.067	


Table S8. The genes involved in I3C accumulation.
Gene	ID	FPKM/W	FPKM/R	FPKM/B	
ASA1	ncbi_106318281	3.787	3.903	3.477	
ASA1	ncbi_106336085	12.91	10.32	14.08	
TSB1	MSTRG.24292	0.23	0.147	0.59	
TSB1	ncbi_106319040	49.687	43.327	53.903	
CYP79B2	ncbi_106301717	2.95	3.11	6.183	
CYP79B2	ncbi_106302779	8.557	6.293	9.473	
CYP79B2	ncbi_106330945	0.52	1.047	1.063	
CYP79B3	ncbi_106337668	0.34	0.5	0.627	
CYP83B1	ncbi_106312180	53.707	46.563	61.103	
GSTF9	ncbi_106328226	353.883	304.803	386.427	
GSTF9	ncbi_106342187	174.807	148.98	174.31	
GSTF10	ncbi_106328484	91.183	69.51	77.03	
SUR1	ncbi_106306456	1.307	1.02	0.44	
SUR1	ncbi_106306752	7.507	4.087	7.153	
UGT74B1	ncbi_106294846	5.247	4.48	6.31	
SOT16	ncbi_106296616	7.907	6.89	9.33	
SOT16	ncbi_106327830	17.597	16.43	18.733	
ESP	MSTRG.17449	1.11	1.757	0.483	
ESP	ncbi_106296341	1.007	1.62	2.63	
ESP	ncbi_106306884	164.543	162.343	118.413	
TGG1	MSTRG.9602	0.067	0.247	0.217	
